# Stress Modulates Illness-Course of Substance Use Disorders: A Translational Review

**DOI:** 10.3389/fpsyt.2014.00083

**Published:** 2014-07-17

**Authors:** Marijn Lijffijt, Kesong Hu, Alan C. Swann

**Affiliations:** ^1^Menninger Department of Psychiatry and Behavioral Sciences, Baylor College of Medicine, Houston, TX, USA; ^2^Human Neuroscience Institute, Department of Human Development, Cornell University, Ithaca, NY, USA; ^3^Mental Health Care Line, Michael E. DeBakey VA Medical Center, Houston, TX, USA

**Keywords:** trauma, stress, addiction, norepinephrine, HPA axis, impulsivity, risk-taking, sensitization

## Abstract

Childhood trauma and post-childhood chronic/repeated stress could increase the risk of a substance use disorder by affecting five stages of addiction illness-course: (a) initial experimentation with substances; (b) shifting from experimental to regular use; (c) escalation from regular use to abuse or dependence; (d) motivation to quit; and (e) risk of (re-)lapse. We reviewed the human literature on relationships between stress and addiction illness-course. We explored per illness-course stage: (i) whether childhood trauma and post-childhood chronic/repeated stress have comparable effects and (ii) whether effects cut across classes of substances of abuse. We further discuss potential underlying mechanisms by which stressors may affect illness-course stages for which we relied on evidence from studies in animals and humans. Stress and substances of abuse both activate stress and dopaminergic motivation systems, and childhood trauma and post-childhood stressful events are more chronic and occur more frequently in people who use substances. Stressors increase risk to initiate early use potentially by affecting trait-like factors of risk-taking, decision making, and behavioral control. Stressors also accelerate transition to regular use potentially due to prior effects of stress on sensitization of dopaminergic motivation systems, cross-sensitizing with substances of abuse, especially in people with high trait impulsivity who are more prone to sensitization. Finally, stressors increase risk for abuse and dependence, attenuate motivation to quit, and increase relapse risk potentially by intensified sensitization of motivational systems, by a shift from positive to negative reinforcement due to sensitization of the amygdala by corticotropin releasing factor, and by increased sensitization of noradrenergic systems. Stress generally affects addiction illness-course across stressor types and across classes of substances of abuse.

## Introduction

Addiction is marked by continued compulsive want for, or use of, mood- and mind-altering substances despite negative consequences or desires to change habits. In 2014–2015, roughly 31 million US adults will be addicted to nicotine [Ref. (1); projected to 2014 US adult population], and 18 and 7.5 million US citizens between ages 12 and 50 will be addicted to, respectively, alcohol and illicit substances [Ref. (2), p. 76]. However, most people who start experimenting with substances will not become addicted, and the question remains why some, but not other, people develop problematic use. Extreme stressful events during childhood or chronic/repeated stressful events during adolescence and adulthood may increase this vulnerability.

Prevalence of childhood trauma (neglect; sexual, physical, or emotional abuse) and post-childhood stressful events is elevated in people with a substance use disorder across classes of substances of abuse, and childhood trauma or post-childhood stress increases risk of developing substance use or use disorders (3–23). Trauma or stress could precede onset of substance use, abuse, or dependence (24), suggesting a potential causal relationship between stress and substance use, although substance use can also predispose to (additional) traumatic events (25).

Adults without psychiatric disorders averaged one or fewer annual major stressful events, compared to three or more among adults meeting criteria for substance use disorder (26, 27), with a potential further gradation in severity of substance use as a function of the number of trauma or stressors people have experienced (28). Post-childhood stressors in people with substance use disorders were mainly financial, legal, social, or occupational (26, 27), suggesting that they may be more chronic and relate in part to consequences of the addiction.

**Figure 1:**
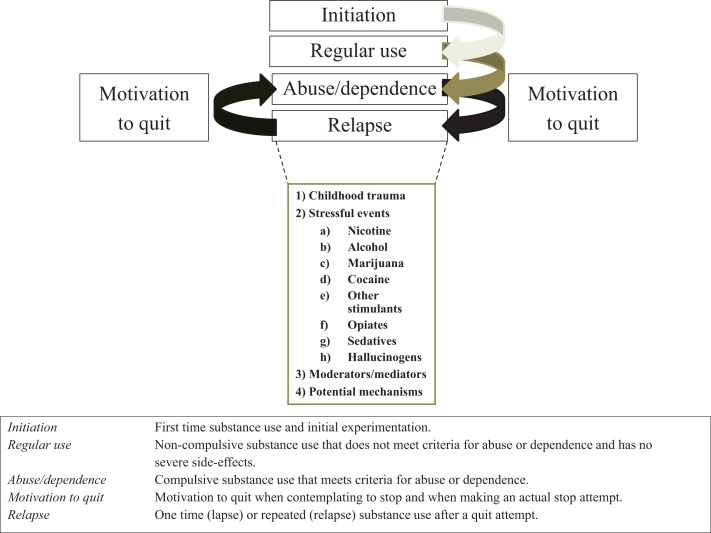
**Stages associated with the illness-course of addiction, definitions or each stage, and topics discussed in this review per illness-course stage**.

Although the negative effects of stress on substance use and use disorders are well documented, to our knowledge a systematic review of relationships between stress and substance use across types of stressors, classes of substances of abuse, or stages of addiction illness-course is lacking. In this review, we first discuss effects of acute and chronic stress on two major stress systems, as well as subsequent effects on motivational and behavioral or emotional control mechanisms. These effects are similar to effects induced by repeated substance use. Next, we review the human literature on relationships between stress and escalation of substance use across types of stressors (childhood trauma and post-childhood chronic/repeated stressful events) and across classes of substances (nicotine, alcohol, marijuana, cocaine, other stimulants, opiates, sedatives and tranquilizers, and hallucinogens) for each of the five stages of addiction illness-course: (a) initiation, or first use of, and experimentation with, a substance; (b) shift from experimental to regular use; (c) escalation from regular use to abuse or dependence; (d) motivation to quit; and (e) relapse. Relationships with potential moderators or mediators (emotional distress, craving, or negative affect) are also highlighted. We end each stage presenting potential mechanisms by which stress might affect illness-course for which we relied on studies performed on humans and animals, and make suggestions about who might be most vulnerable to escalation. Stages and topics per stage are presented in Figure 1.

## Stress and Drugs: Overlapping Mechanisms

### Acute stress and substance use

Stress refers to any unpredictable or uncontrollable event that “exceeds the regulatory capacity of an organism” (29), and that threatens or could threaten an organism’s physical or psychosocial integrity (30). Acute stress temporarily activates the hypothalamic-pituitary-adrenal (HPA) and the sympathetic-adrenal-medullary (SAM) axes, respectively increasing glucocorticoids (cortisol in humans and corticosterone in rats and mice) and norepinephrine (31), subsequently activating or inhibiting other mechanisms so that the body can cope with the stressor (32).

The paraventricular nucleus (PVN) of the hypothalamus gathers information about stressors via neuronal projections from brainstem nuclei, sensory cortices, amygdala, and the prefrontal cortex, activating the HPA axis by increasing release of corticotropin releasing factor (CRF) and arginine vasopressin (AVP) (33–36). CRF, potentiated by AVP, triggers release of adrenocorticotropic hormone (ACTH) by the anterior pituitary, which in turn signals the adrenal cortex to increase glucocorticoid availability; glucocorticoids regulate the stress response by signaling the brain to inhibit HPA axis activation (33, 37–39). The HPA response is regulated in part also by the hippocampus (40–42), the medial prefrontal cortex (43, 44), and the amygdala (45). Deactivation or diminished functioning of the cortisol feedback loop, the hippocampus, or anterior cingulate cortex and increased activation of the amygdala have been related to impaired reactivity of the HPA axis in response to stressors (41, 46). Substances of abuse have similar effects on HPA axis activation as stressors (47), suggesting shared mechanisms.

Dopaminergic mechanisms, including the ventral tegmental area (VTA) and the nucleus accumbens (NAc), are thought to mediate the rewarding and addictive aspects of drugs across all classes or substances of abuse. In a comprehensive review, Leyton (48) showed that all substances of abuse, administered acutely, increase striatal extracellular dopamine, and that increased dopamine correlated with increased pleasure or motivational/rewarding effects of substances, increased sensitivity to drug-related stimuli, and compulsive want to consume substances (48). The two latter effects occur in later stages of addiction illness-course and could relate to neural adaptations of striatal mechanisms due to repeated stimulation of dopamine receptors, leading to sensitization of reward systems to drugs and drug-related stimuli (incentive sensitization) which can persist for years (49). Sensitization can be measured by excessive motor or motivational responses to doses of substances that prior to (escalation of) use did not evoke these reactions. Sensitization could increase risk for relapse even years after use of a substance, and could accelerate development of addiction-like patterns to other drugs due to cross-sensitization (48).

Stress has comparable effects on striatal dopamine systems as substances of abuse. CRF increases dopamine release in striatal areas by binding to CRF-2 receptors on glutamatergic cells in the VTA, subsequently activating neurons containing dopamine (50, 51). In humans, acute uncontrollable psychological stress decreased [11^C^]raclopride binding potential in the ventral striatum, including the ventral putamen and NAc (52), indicating increased striatal extracellular dopamine under stress. This allows stress to sensitize reward systems, resulting in cross-sensitization between stressors and addictive drugs (53). More pronounced cortisol reactivity to stressors related to a more pronounced reduction in [11^C^]raclopride binding, suggesting a more pronounced effect on reward mechanisms in people with higher stress reactivity (52).

Stressors also activate the SAM axis, in part by CRF signaling, increasing release of norepinephrine from the locus coeruleus (54–56). Stress-induced norepinephrine release elevates norepinephrine availability in the frontal cortex (57, 58), disrupting prefrontal functioning by flooding α1-adrenergic receptors (54), and increasing impulsive responding in people with higher norepinephrine reactivity (59). Stress-induced norepinephrine release could negatively affect emotion, motivation, and control functions (54, 59, 60), induce habitual rather than goal-directed actions (61), and bias processing of motivational information (62–64).

### Childhood, chronic or repeated stress, and substance use

Stressors and substances of abuse both activate stress mechanisms (47). Repeated or chronic HPA axis activation by repeated stress or substance use can lead to impaired stress responses, including blunted (impaired HPA axis activation) or prolonged responses (impaired HPA axis inhibition), and a lack of HPA axis habituation to repetition of the same stressor (32). Repeating the same stressor attenuates release of ACTH and cortisol, whereas norepinephrine and epinephrine do not habituate with repetition (31). Trauma or stressful events could increase HPA axis reactivity in humans, especially in those with more symptoms of depression (65).

Chronic stress in rats reduced medial prefrontal lobe volume (66), possibly by decimating dendritic spine density of pyramidal neurons in the medial prefrontal cortex (67), an area implicated in regulating the human HPA stress response (33) and human emotional, cognitive, and behavioral control (68, 69). Similar effects on the prefrontal cortex have been found for childhood trauma, impairing prefrontal functioning (70).

Furthermore, early trauma, chronic stress, and prolonged substance use can further intensify sensitization of dopaminergic reward systems by enhancing synaptic strength of excitatory synapses on VTA and NAc dopamine cells (71). Amygdala activity could be increased also by repeated stress, which could be accompanied by increased anxiety and fear-induced behavior (32), and by a CRF-induced shift in later stages of addiction from positive reinforcement to negative reinforcement in which substance use is driven by motivating effects in initial stages of substance use, but by withdrawal-related effects in later stages of illness-course (45, 72). This shift is accompanied by substance-induced HPA axis activation in early stages followed by withdrawal-induced HPA axis activation in later stages of addiction illness-course (45, 72, 73). Finally, the noradrenergic response may become sensitized or more reactive by repeated or chronic activation of the locus coeruleus by stress or substances of abuse (37, 74, 75), potentially increasing the negative effect of stress-induced norepinephrine release on prefrontal functioning (55).

Thus, trauma and stress occur earlier, are more common, and could be more chronic among people who use substances. Early, chronic or repeated HPA or SAM activation by stress or substances may predispose people to addiction by changing motivational, emotional, and behavioral systems. Next we review the human literature on relationships between stress and substance use for each of the five stages of addiction illness-course: (a) initiation, or first use of, and experimentation with, a substance; (b) shift from experimental use to regular use; (c) escalation from regular use to abuse or dependence; (d) motivation to quit; and (e) relapse. We also discuss potential moderators or mediators, present mechanisms by which stress might affect transition to illness-course stages, and make suggestions who might be most vulnerable to escalation.

## Effects of Trauma or Stress on Stages of the Addiction Cycle

### Opportunity, initiation, and experimentation

For some people, addictions start at the very first opportunity to use (or try) a substance, which is often in childhood or early adolescence (76, 77). Daily in the United States, roughly 6400 people use tobacco for the first-time, 12,600 people initiate use of alcohol, and 7900 people initiate use of illicit substances; about half of these people are between 12 and 18 years old (2).

#### Childhood trauma

Childhood trauma has been associated with increased opportunities to try substances (78) and has been related to an elevated risk to initiate use of cigarettes, alcohol, marijuana, cocaine and other stimulants, opiates, and sedatives during childhood, adolescence, and adulthood (28, 78–86), and to earlier age of onset for use of alcohol, stimulants, opiates, or sedatives than people who did not experience (13, 87–90) or who experienced less severe childhood trauma (91). These risks remained elevated into adulthood (82), regardless of sex (87), in part due to greater likelihood of living in disadvantageous neighborhoods as adults (92). By contrast, some studies found no relationships between childhood trauma and lifetime use of marijuana or alcohol in adolescents (93), use of marijuana among adolescents and young adults (94), or earlier initiation of substance use across various classes of substances (25). This latter study reported that, instead, substance use may predispose people to trauma due to elevated risk-taking (25), although this may only hold true among a subsample of adolescent and adult users.

#### Stressful events

Post-childhood stress or stressful events have also been related to (earlier) initiation of various classes of substances. Stressful events can include death or serious illness of a family member or close friend, relocation, being fired or laid off, a financial crisis, trouble with a boss or coworker, separation, divorce or break-up, serious interpersonal strain with a friend, neighbor or relative, contact with law-enforcement, or being a victim of violence (95). Some studies also addressed uncontrollable daily hassles, or problems at home, school, or work. We will present relationships for specific substance classes where possible. However, studies have frequently pulled classes together under the heading “(illicit) drugs.”

School- or family-related stress has been associated with an elevated intention to start smoking among adolescents who did not smoke (5), and among adolescents smoking initiation was predicted, in part, by parental job loss (96), increased problems at school (97), lower parental social economic status (98), and elevated perceived stress (7, 99). Elevated stress has also been related to earlier onset of smoking in adolescent girls (16).

Alcohol initiation was predicted, in part, by transitioning during adolescence from a single-parent household to a household with a stepparent (100), and smoking and alcohol use increased population-wide after major national or local disasters [reviewed in Ref. (101)], suggesting increased initiation or relapse. Furthermore, a history of more stressful life events may relate to an earlier onset of first alcohol use (102), although this was not found for recent (6-month) life events (103). This discrepancy could be due in part to a modulation of stress-related early onset of alcohol use by polymorphisms of genes encoding for CRF receptors (102); combining polymorphisms may cancel out effects.

Earlier initiation of marijuana use was predicted by more daily hassles and lifetime stressful events in 14–24 year old participants (94), and paternal weekly drinking to intoxication (parental report) when participants were aged 11–12 (104).

A study of initiation of “illicit” drugs reported elevated risk among 10–16 year old participants when participants were growing up in a disadvantageous neighborhood, which has been designated as a chronic stressor (105), and recent (6-month) stressful events reported by 12–14 year old adolescents, but not lifetime stressful events reported by their parents or self-reported negative affect, predicted earlier onset of use of marijuana, crack, cocaine, heroin, or amphetamines (measured as one global category) (103), whereas more relocations before age 16 related to early initiation of using marijuana, crack, cocaine, hallucinogens, or prescription drugs (generally sedatives) among 18–35 year old adults (106). Finally, homelessness, a history of incarceration, unstable housing, or trading sex for drugs related to a higher risk to initiate injecting any drugs (83, 84), methamphetamine (82), or cocaine (107) in young adult drug users previously naïve to injecting the corresponding substances, and about a quarter of adult inmates reported first-time heroin use in a UK prison (108).

Thus, various chronic or repeated stressors predispose adolescents and young adults to start experimenting with various classes of substances, and to initiate use at an earlier age. However, types of stressful events that have been related to initiation appear inconsistent across studies and substance classes.

#### Moderators or mediators

Increased stress, more emotional distress, or expected stress relief were the main reasons for smoking initiation among male and female adolescents (109, 110), and elevated anger, nervousness, anxiety, depression, shyness, or dissatisfaction with life increased risk to initiate use of nicotine, alcohol, or marijuana among adolescents aged 9–15 (111, 112). However, anxiety and depression in 12 year olds was not associated with earlier onset of alcohol or drug use (103). Therefore, stress-induced or general emotional distress may predispose adolescents to start using nicotine, alcohol, or marijuana, perhaps because of expected relief of emotional distress or negative affect, but it did not shift first-time use to an earlier age.

#### Summary

Childhood trauma and post-childhood chronic or repeated stressful events increased the risk to initiate use of substances, and to start first-time use at an earlier age across substance classes. Emotional distress or negative affect was related to elevated risk of initiation, but not earlier age.

#### Potential underlying mechanisms

Stress may not affect initiation by influencing motivational systems. Instead, stress might affect trait-like factors, including risk-taking, decision making, and behavioral control, or the belief that substances could relieve stress (109, 110).

Risk-taking is elevated in early adolescence compared to childhood and late adolescence (113). Higher risk-taking and sensation seeking in early adolescence enhanced the risk of early onset of alcohol use (114). Risky actions have been related to increased activity of the ventromedial prefrontal cortex, whereas safe actions have been related to activation of the lateral prefrontal cortex (115). Expert advice, for example by a parent or teacher to not use drugs, could diminish risk-associated activity of the ventromedial prefrontal cortex or increase safe-associated activation of the lateral prefrontal cortex (116). However, stress could increase risky actions (117), in particular in people with elevated stress reactivity of the HPA axis or noradrenergic nuclei (118). This may be due in part to stress-related impaired decision making and behavioral control associated with inhibition of prefrontal functioning (55) by stress-induced enhanced occupancy of dopamine D1 and α1-adrenergic receptors in prefrontal areas (59). Norepinephrine can remain elevated throughout the duration of stressors (119), with an extended elevation after the end of the stressor among people with a history of childhood trauma (75), suggesting an increased risk of stress-induced poor decision making. Finally, some may have a bias that substances could relieve stress (109, 110).

Probability of poor or risky decisions may be increased during or following stress, including periods of emotional distress. However, people with a history of childhood trauma are particularly vulnerable to making poor decisions due to increased reactivity of the HPA and SAM axes (74, 75) and to impaired functioning of the dorsolateral and ventromedial prefrontal cortices (70), which regulate cognitive, emotional, and behavioral control (68, 69).

**Figure 2:**
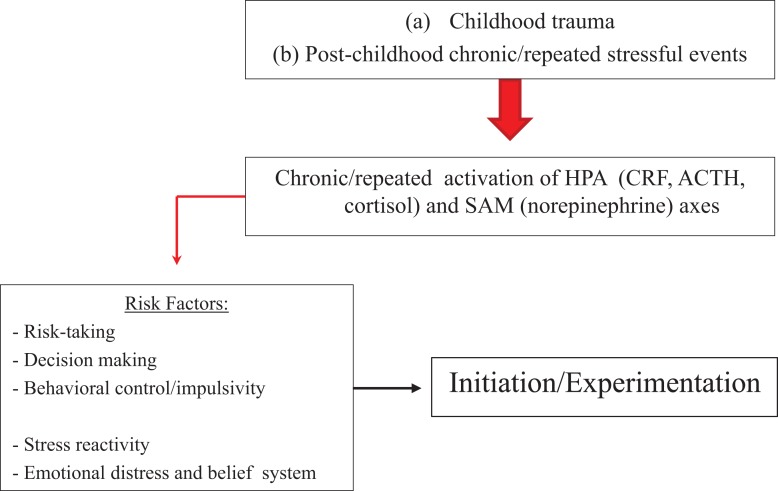
**Model on how stressors may affect substance initiation or initial experimentation**. Stressors affect HPA and SAM axes, which can increase risk-taking, and impair decision making and behavioral control. At particular risk are people with trait-like high risk-taking, impaired decision making, poor behavioral control or high impulsivity, increased stress reactivity, or elevated emotional distress with a belief that substances diminish negative affect.

Figure 2 presents the relationship between trauma or stress and substance use initiation, which may relate to stress-induced effects on trait-like factors such as risk-taking, decision making, and behavioral control or impulsivity. People with trait-like elevated stress reactivity, negative affect and the belief that substances can improve mood or relief stress, increased risky behavior, poor decision making skills, or impaired behavioral control and higher impulsivity may be at particular risk for stress-induced initiation.

### Shift to regular use

#### Childhood trauma

Nelson et al. (120) studied effects of childhood sexual abuse in adults on any use, regular use, or abuse/dependence of nicotine, alcohol, marijuana, cocaine, stimulants, sedatives, or opioids, and found that regular use of nicotine, but not alcohol, and any use of marijuana, cocaine, stimulants, sedatives, or opioids (regular use was not assessed for illicit drugs) were predicted by a history of childhood sexual abuse. Moreover, the risk to use any licit or illicit drugs increased linearly with accumulative childhood trauma (4).

#### Stressful events

School- or family-related perceived stress or stressful events elevated risk of smoking, alcohol use, or use of any illicit substances among adolescents (7, 8, 10, 11). Seventy-two percent of adolescents and young adult smokers reported they progressed from experimentation to regular use because they felt stressed (28). Similarly, perceived stress (121), more problems in school (97), and elevated family poverty (122) increased risk to shift from experimental to regular smoking in adolescents. By contrast, self-reported daily hassles and stress reactivity (emotional distress and increased cortisol) to an uncontrollable psychosocial stressor did not relate to smoking behavior at time of the experiment or at 1-year follow-up in adults who smoked fewer than 20 cigarettes per week (123).

One study looked at potential causal relationships between stress and smoking or drinking by studying the effects of exam stress in undergraduates on use of nicotine, alcohol, and caffeine (124). This study did not assess symptoms of abuse or dependence, but based on self-reported average daily cigarette (four cigarettes), and weekly caffeine (four units) and alcohol use (four to six units), subjects appear to be primarily non-dependent regular users combined with people meeting criteria for nicotine or alcohol abuse (binge drinkers). Substance use was assessed 1 month before and during an exam period; substance use in a control group was assessed twice in non-exam periods with a delay between both assessments matching the delay between assessments in the exam group. Compared to the non-exam baseline, the exam period was associated with elevated emotional distress and negative affect, as well as increased daily use of cigarettes (about 12 on average), elevated weekly use of caffeine (about 11 on average), but no change in alcohol consumption. No change was found for substance use in the control group (124). The increase in caffeine use in the stress-group may be explained by use for its stimulating effect, whereas increased cigarette consumption (3× baseline) may be explained in part by increased stress in undergraduates who used substances regularly but did not necessarily meet criteria for abuse or dependence. Another study investigated relationships between acute stress and substance use in a laboratory setting, showing that, compared to a neutral manipulation, uncontrollable psychosocial stress increased post-manipulation consumption of alcohol, but not a placebo drink, in non-dependent social drinkers with a family history of alcohol use disorder (125).

By contrast, for marijuana use major life stress, including parental death before participants were 15 years old, and parental drug problems, *protected* against frequent use among 14–24 year old participants who had initiated marijuana use, whereas daily hassles had no effect (94), suggesting major stressors may protect against, or have no effect, on the transition from experimental to regular marijuana use.

Thus, chronic or repeated stressors could escalate people to start using substances more regularly after a period of experimentation, and to increase intake among regular users.

#### Moderators or mediators

Stress-induced emotional distress in smokers with no or low dependence to nicotine did not relate to changes in smoking behavior during stress-induction or at 1-year follow-up (123). On the other hand, transitioning from experimental to regular smoking was predicted by low self-esteem (8, 126, 127) and increased negative affect (10, 128).

#### Summary

Childhood trauma appears to increase risk to transition from experimental to regular use of nicotine, marijuana, cocaine, other stimulants, opiates, or sedatives. Stressful events could facilitate the transition to regular nicotine and alcohol use, but protect against this transition for marijuana. There is little information on potential relationships between post-childhood chronic or repeated stress and progression from experimental to regular use for cocaine, other stimulants, sedatives, opiates (including heroin), and hallucinogens.

#### Potential underlying mechanisms

By contrast to effects of stress on initiation, effects of stress on transitioning to regular use and to abuse/dependence could relate to mutual effects of stress and substances on motivational mechanisms, potentially combined with effects of stress on trait-like mechanisms discussed in the previous section. Progression from experimental to regular use may relate to stress-induced increase in reward motivation of lower doses of substances, rapid escalation to regular use, and increased dose and frequency of intake. These effects could relate to cross-sensitization between stress and substances of abuse, accelerating the increase in motivational value of substances. Individuals with high trait impulsivity may be at particular risk.

Stress could accelerate progression to (concentrated) regular use. Substance-naïve adult rats were exposed to an aggressive rat or a tail-pinch 4–7 days prior to first-time exposure to cocaine (129–132) or methamphetamine (133). Compared to non-stressed control animals, those that had encountered stress showed increased cocaine or methamphetamine self-administration 3–7 days after first-time use even though stressed and control animals initially had similar use levels (129–132). This increase in self-administration may reflect an increased tendency of stressed animals to binge (129). Interestingly, cocaine self-administration also increased in stimulant-naive rats that had to witness another rat receiving foot shocks (which is an emotional stressor), whereas those that received the shocks did not show an increase in self-administration shortly after first-time use (134), suggesting emotional stressors are more potent than physical stressors in inducing escalation in experimental users to more regular use.

Intermittent use of substance, for example during weekends, may mark substance use in the stage after experimentation, and appears to be the most efficient way to initiating sensitization of motivational systems (49, 135). In animals, sensitization is reflected by increased motor activation induced by a small dose of a substance after animals have abstained from repeated intake, compared to motor activation at initial intake, and increased preference for a part of the cage that signifies substance availability (conditioned place preference) after repeated substance self-administration. Sensitization relies, in part, on dopaminergic striatal mechanisms (49, 135). Stress (and all drugs of abuse) affects dopaminergic reward and motivation systems (48, 50, 51), and increases sensitization and cross-sensitization to substances and additional stress (53). After initiation, repeated, but erratic, use of substances that are readily available allows this system to become increasingly more sensitized to drugs and stress, and, via Pavlovian learning in later stages of addiction illness-course, to internal or external cues signifying the potential use of a substance (e.g., a neon sign) (49, 135).

In healthy, drug-naïve men, sensitization of dopamine systems, measured by motor responses and striatal dopamine receptor occupancy, can be induced after only three administrations of amphetamine, and persist for at least 1 year after the last administration (136). Furthermore, men with elevated trait impulsivity, a marker for people with a substance use disorder irrespective of class of preferred substance (137), were more prone to sensitization than men with lower trait impulsivity (136).

Stress could also elevate motivational value of substances among experimenters or non-dependent regular users. Among healthy adult men, acute administration of d-amphetamine after stress induction was rated as more pleasurable by those who reported more stress-induced distress than among those who reported less distress (138). This effect of stress on motivation has also been shown in animals. Nicotine-naïve rats stressed 24 h earlier acquired place preference at lower doses of nicotine than non-stressed rats (139); the potentiating effect of stress on motivation, but not on acquiring place preference itself, was diminished after pre-treatment with a CRF-1 receptor antagonist. Similarly, stress prior to escalation to regular use in morphine-initiated mice resulted in repeated self-administration of lower dosages irrespective of whether mice preferred low or high dosages prior to stress induction. This effect was shown for mice that were stressed by witnessing another mouse receiving foot shocks (inducing emotional stress), but not for mice that received foot shocks (140). These outcomes suggest increased sensitivity to the motivational effects of drugs after childhood trauma or chronic/repeated stress, reducing the dose required for maintaining substance use, which could be partly mediated by CRF.

**Figure 3:**
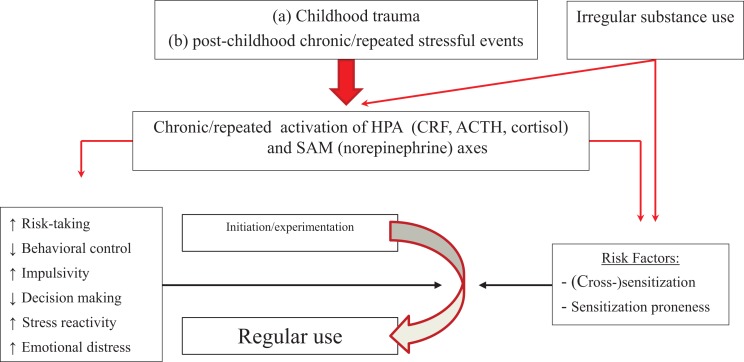
**Model of how stressors, in conjunction with initial irregular substance use, may affect progression from experimentation to regular use**. Repeated stress can cross-sensitize motivational systems to the motivating effects of substances of abuse, which can be further sensitized by irregular substance use (right side of picture). This might operate in parallel with effects of stress on trait-like factors (left side of figure). People with high trait impulsivity, who are more prone to sensitization, may be at particular risk.

Figure 3 presents the effects of stress system activation by stressors and by irregular use of substances of abuse on transitioning from experimental to regular use, which could be due initially to stress-induced sensitization and cross-sensitization of motivational systems, especially in people with high trait impulsivity or increased stress-induced negative affect. This may lead to increased motivational significance of lower doses, faster escalation to binging, and elevated satisfaction, pleasure, or reward from substance after stress (138, 141, 142), thus potentiating positive reinforcement. The potential interaction between stress and sensitization could exist in addition to the effects of trait-like factors described in the previous section.

### Escalation to heavy use, abuse, and dependence

#### Childhood trauma

Childhood trauma increases risk to develop a substance use disorder (12–15, 17–20, 23, 78, 86, 93, 143, 144), including for nicotine, alcohol, marijuana, stimulants, sedatives, and opioids, but not cocaine (120). In addition, a history of childhood sexual, physical, or emotional abuse was related to heavier or more frequent alcohol or substance use in adults across substance use disorders (22, 88, 93), although other studies did not find this (89). Risk to use any licit or illicit drugs (4) and the severity of heavy drinking (143) and nicotine dependence (93) increased with severity of childhood trauma. Interestingly, the relationship between childhood trauma and substance use disorder could be mediated by increased trait impulsivity (22) or subsequent stressful events in adolescence or adulthood (145, 146), suggesting that the risk to develop a substance use disorder among people with a history of childhood sexual abuse or other trauma is greater in those with higher impulsivity or those who encounter additional stressful events.

#### Stressful events

In adult smokers, longer hours of work, tendencies of workaholism, perceived lack of influence, increased stress, and less job satisfaction related to smoking more (147–149), and to meeting more symptoms for nicotine dependence (150). Relationships between stress and nicotine intake have also been studied in laboratory settings. In adult smokers, administration of psychosocial stressors resulted in participants smoking more cigarettes (151) or taking more puffs (152) directly following the stress manipulation compared to a neutral control manipulation or compared to a control group. These outcomes have been related to stress-induced diminished self-control to resist smoking cravings during acute withdrawal (153), suggesting a relationship between stress, craving, and poor behavioral control. However, others have reported that stress manipulations in the laboratory resulted in only a non-significant trend to smoke after stress exposure compared to a neutral manipulation (154), and that actual stressful events did not predict cigarette use (155).

For alcohol, living in a disadvantageous neighborhood during adolescence predicted developing alcohol use disorder (156), and more lifetime stressful events predicted increased risk of binge drinking and drinking more per occasion, suggesting alcohol abuse, in 15 year old participants (157). Similar relationships have been reported also among adults. Elevated self-reported psychosocial stress increased risk of binge drinking and regular drug use (158), and more past-year stressors related to more past-year daily alcohol use, with approximately six daily alcoholic drinks in those who reported having encountered six or more past-year stressors (159). This relationship was stronger in people with alcohol use onset before age 14 than in people with later onsets of use (159). This latter effect might be modulated by a polymorphism of a gene encoding for corticotropin releasing hormone receptors (157).

Elevated stress among active duty service members was related to heavy drinking, smoking, and use of illicit substances (6). Furthermore, members of the reserves or National Guard, but not active personnel, deployed to war-zones who participated in active combat were at increased risk to develop post-deployment heavy alcohol use and alcohol-related problems compared to members not exposed to active combat (160). On the other hand, risk of developing post-deployment binge drinking was increased in active service members irrespective of active combat (160). Developing post-deployment heavy drinking and alcohol abuse was strongest in those who had experienced threat of bodily injury or death during active combat (161). This suggests that stress exposure could increase the risk of consuming more alcohol among previous regular users consistent with reviews on the effect of stress on continuation of alcohol use (162). On the other hand, some studies reported no relationships between stressful events and drinking patterns: recent life stress did not predict drinking patterns when also accounting for childhood trauma (143) or sex (146); this latter study found stressful events to relate to more heavy drinking in men, whereas in women more heavy drinking related to stressful events only in those with a history of childhood trauma.

For marijuana, more major stressful events, particularly major financial problems, predicted transition from non-dependent to dependent use among 199 young frequent marijuana users (163), replicating other studies showing a relationship between marijuana dependence and major financial problems (94, 164). Increased risk for this transition was also predicted by parental death prior to age 15 (94) or parental divorce/separation (165), although this latter finding has not been reported by other studies (166).

In a large sample of 12–17 year old adolescents, risk of drug (across classes) dependence was predicted by living in a disadvantageous neighborhood or by low family income (156). Unfortunately, no specific substance classes other than alcohol were assessed. Disadvantageous neighborhoods, considered a chronic stressor (105), could provide increased opportunity to initiate (77) and maintain use. Finally, a history of incarceration, unstable housing, and trading sex for drugs have been related to more frequent cocaine injection among adults injecting cocaine (107).

Thus, chronic or repeated stressors could escalate people who use substances regularly to develop substance dependence, in particular in those who started use in early adolescence or in those with poor behavioral control.

#### Moderators or mediators

Stress in laboratory settings or in daily life increases craving and/or emotional distress in people who, at time of testing, met criteria for substance use disorder and were actively using nicotine (153, 154, 167–176), alcohol (177), marijuana (39, 178), cocaine or other stimulants (179–183), or opiates (184, 185). These stress induced increases in craving and emotional distress have been reported often, although some studies reported no effect of stress on craving, potentially because the stressors were too mild (moderately difficult arithmetic task) (184) or because of sex differences (183). The latter study suggests different effects of stress on craving in men and women, although findings have been inconsistent: effects of stress on craving could be stronger in women than in men addicted to nicotine (171) or cocaine (183), although high progesterone may partially protect against stress effects on craving (186). On the other hand, no difference was found for the effect of stress on craving or emotional distress in men and women with cocaine use disorder who were abstaining, whereas the neuroendocrine response to stress was enhanced in men relative to women (187). Thus, stress could induce emotional distress, negative affect, or craving.

Emotional distress, negative affect, and craving can subsequently lead to substance use. Cigarette use was predicted by increased subjective feelings of depression, negative affect, and perceived social stress (155). This is consistent with findings that the transition from experimenting with smoking in adolescence to nicotine dependence in young adulthood related to more symptoms of depression and anxiety (128). Subjective distress, including symptoms of anxiety or depression, in adolescents also predicted more frequent marijuana use (165, 188) and transition to marijuana dependence (189), although an effect on transition was not found by others (165). Finally, across adult homeless women, symptoms of depression correlated with symptoms for drug use disorder and problems due to drug use (190).

Stress-enhanced craving or emotional distress could result in continued substance use due to the belief that substances can help regulate these emotions. Indeed, adult smokers reported stress relief was an important motive to continue smoking (155, 191, 192), and elevated negative affect was related to using more tobacco (193). The relationship between stress and use of nicotine, alcohol, or marijuana to regulate distress or negative affect was stronger in those with increased anxiety sensitivity (194) or in people with a family history of alcohol problems (195). Finally, in a sample of people who used marijuana 2–7 days per week, suggesting abuse or dependence, lower self-reported distress tolerance related to higher scores on a questionnaire that marijuana was used to relieve feelings of stress or negative affect (196).

#### Summary

Childhood trauma and post-childhood stressful events could escalate recreational, non-problematic, substance use to use that meets criteria for substance use disorder marked by compulsive intake at high personal and social costs. This could represent a direct effect of stress or indirect effects of stress via craving or emotional distress. In addition, during dependence stress and emotional distress experienced at acute withdrawal may precipitate new intake (45, 197, 198). Acute withdrawal has been shown to increase craving, feelings of depression, irritability or anger, restlessness, and to impair concentration in smokers over a 24-h period of abstinence (170, 197, 199); these symptoms are also observed in non-abstinent people with substance use disorders who experience stress (198). This cycle makes it more difficult to quit using, even in someone who wants to stop. There is only limited information on the effect of childhood trauma on severity of a substance use disorder for specific classes of substances. For stressful events, information is limited for effects on risk and/or severity of marijuana, stimulant, opiate, sedative, or hallucinogen use disorders, whereas the relationship between stress and increased severity of nicotine addiction is inconsistent across studies.

Potential mechanisms underlying stress-induced escalation from regular non-dependent use to dependence could resemble mechanisms underlying stress-induced relapse. Therefore, these mechanisms will be discussed below, following the overview on relations between stress and relapse. Mechanisms could involve (a) further sensitization of reward mechanisms by stress and substances, with (b) a transition from positive reinforcement during regular use to negative reinforcement during dependence and relapse, and (c) a concomitant change in HPA axis activation.

### Quitting and motivation to quit

Most people with a substance use disorder quit or cut back at some time (200–203). However, stress reduces the likelihood of quitting, potentially by reducing the motivation to quit relative to the motivation to continue drug use. Elevated work or financial stress has been related to a reduced likelihood to cease smoking (149, 204–206), and young adults were less likely to quit using marijuana when they had a history of long-term unemployment (207). In young adults with a history of injecting drugs, homelessness (208), or a history of incarceration (209) predicted lower motivation to cease drug use. Research in smokers suggests motivation to quit could be diminished in some users due to an inability to regulate stress or emotional distress without the help of mind-altering substances (155, 191, 192).

Although limited in scope, these studies suggest that stress reduces likelihood and motivation to quit, in particular in people who use substances to find relief from stress, emotional distress, or acute withdrawal. More research is needed to determine whether and how stress could affect the motivation to quit, what types of stressors are the most relevant to motivation, how the motivation to quit is related to childhood sexual abuse or other trauma, and whether stress–motivation relationships may be similar across classes of substances and age groups.

### Lapse or relapse

#### Childhood trauma

Individuals with a substance use disorder who want to stop using commonly lapse or relapse at least once (210–216). Risk of (re-)lapses could be increased, or be more frequent, in women, but not in men, with childhood trauma and a cocaine (217) or non-differentiated substance use disorder (218, 219). More traumatic events related linearly to a higher risk for a post-treatment relapse (213), potentially due to lower improvement during treatment on outcomes of substance abuse or continued exposure to new traumatic events among people with a history of childhood trauma (218). On the other hand, in people who were treated for alcohol use disorder, a reported increase in relapse risk with childhood trauma disappeared after controlling for demographic and psychiatric variables (220). This latter finding is in line with other studies that found no significant relationship between (childhood) trauma and post-treatment relapse in adults (221, 222).

#### Stressful events

Among smokers, psychosocial stressors related to an increased failure to quit (223), and relapse risk was higher in people who had recently changed residency or who had major financial problems (206). These relationships may involve specific motives why to smoke, and be modulated by stress tolerance: smokers who smoked to regulate stress relapsed more often than people who smoked for other reasons (224), and stress tolerance was diminished among active smokers with a history of relapsing within 24 h after starting a quit attempt compared to smokers with a history of abstinence that lasted at least 3 months (225). Stress tolerance was tested by allowing subjects to stop stress-provoking experiments, which were either a moderately difficult calculation test containing elements of a working memory task or administration of air with additional carbon dioxide (225). These findings were not replicated in a different sample of active smokers (211), although a significant relationship was found between low stress tolerance and early lapses when people were asked after testing to stop smoking for 28 days (211).

A recent narrative review of 18 studies published between 1988 and 2010 included 10 studies on stress and relapse in people with alcohol use disorder (226). Stress was defined as major stressful events or daily hassles. More negative life events related to increased risk of relapse, although findings were inconsistent across studies, potentially due to methodological differences (226). On the other hand, studies not included in the review found that more stressful events increased relapse risk in adults at 3-month and 1-year follow-up (227), and a recent study in a nationally representative sample of non-institutionalized US adults found that risk for a relapse at 3-year follow-up among people with a history of alcohol use disorder who were in full remission at baseline assessment was higher after a divorce or separation in the year before baseline assessment (228).

In another study in the same nationally representative sample of non-institutionalized US adults, relapse risk at 3-year follow-up in adults with a history of marijuana use disorder who were in full remission at baseline assessment was increased with more self-reported stressful events in the 12-months prior to baseline assessment (229). Similarly, more major stressful events during the 3-months preceding follow-up predicted relapse among 304 people with primarily cocaine use disorder who had been abstinent for at least 3 weeks (230). The narrative review mentioned in the last paragraph (226) that included the latter study, also reported increased relapse risk with more negative stressful events among people abstaining from opiates, cocaine, or nicotine, although two other studies (one with similar samples of people using opiates, cocaine, or nicotine and the other with people with opiate use disorder who were on methadone treatment) reported no significant relationship between relapse and stressors.

Finally, for studies combining substance classes, increased self-reported stress in adolescents admitted to a substance abuse treatment clinic predicted earlier treatment discontinuation (231), and adults in active treatment with a history of family violence tested positive on drug screens more often than those without history of family violence (232). Among adult American Indian women, relapse risk 6 and 12 months after treatment was predicted in part by family conflict at intake (233), and risk of substance use at 1-year follow-up was predicted by baseline perceived stress among people convicted in drug court (234), and by a history of incarceration or homelessness in people injecting drugs (235). Similar to relapse risk in smokers, for people using other substances lower stress tolerance measured as the request to stop stress-provoking tests related to shorter time to relapse of the most recent quit attempt (212). Finally, more life stress assessed post-treatment moderated a relationship in adolescents between stress-induced poor behavioral control and increased relapse risk during 3 and 6 month follow up (236).

Thus, chronic or repeated stressors could instigate relapse, in particular in those people with elevated stress sensitivity or poor behavioral control.

#### Moderators or mediators

Stress in laboratory settings or in daily life has been related to elevated craving and/or emotional distress in people with a history of a substance use disorder who had early or prolonged abstinence from using nicotine (237–239) alcohol (240–244), marijuana (245), cocaine or other stimulants (210, 246–250), and opiates (249, 251) [however, see Ref. (184) for no effect in people with a history of opiate use disorder who abstained from using opiates]. Elevated craving [e.g., Ref. (210, 237, 242, 252, 253)] and emotional distress or negative affect [e.g., Ref. (179, 237)] predict relapse, although specific pathways may differ between adolescents and adults (215). In adolescents, relapse could relate to using a substance to enhance a positive state combined with environmental pressure (path 1, reflecting positive reinforcement) or (in part) to cope with negative affect and stressful situations (path 2, negative reinforcement). In adults, relapse could relate to using a substance to cope with negative affect combined with an inability to regulate urges (path 1, negative reinforcement and poor self-control) or to social or environmental pressures (path 2) (215).

#### Summary

Childhood trauma and post-childhood chronic or repeated stressful events could increase relapse risk or risk for a more severe relapse, but results are not consistent across studies and seem to be limited mostly to nicotine and alcohol. More research is needed on the effects of childhood trauma or post-childhood stressful events on risk and severity of lapse or relapse for individual classes of substances of abuse. Potential relationships between stress and relapse may be moderated by stress-enhanced craving or emotional distress.

#### Potential underlying mechanisms

Escalation to compulsive use and risk for relapse could in part relate to (a) increased incentive sensitization of reward systems resulting in increased strength of the motivational effects of drugs (and drug-related stimuli) (49, 135), (b) increased reliance on substance use to avoid negative symptoms of early withdrawal (45, 72); and increased sensitization or reactivity of norepinephrine, impairing control functions (54, 59, 60), and increasing salience of motivational cues (62–64). These mechanisms may explain increased compulsive use of substances even while people with a substance use disorder show a diminished liking of the substances.

Previous or continued stress and continued use of substances contribute to further sensitization of dopaminergic reward mechanisms, uncoupling liking drugs from wanting drugs (49, 135). This increase in sensitization could further strengthen the learned link between substances or substance effects and (prior non-rewarding) environmental stimuli, inducing priority processing of (attentional bias), and more pronounced salience attribution to, substance or substance cues than to naturally rewarding stimuli (e.g., food or intimacy). This was elegantly illustrated by Versace et al., who showed, using evoked potentials (254) or functional imaging (255), that people with nicotine use disorder have elevated brain activation to stimuli signifying smoking or cigarettes, indicating increased allocation of attentional resources to motivational information. However, people could be clustered according to equal attention allocation to smoking and naturally rewarding stimuli, versus diminished activation to naturally rewarding stimuli compared to smoking-related information. The latter group had shorter latencies to relapse than the former group. This effect might be stronger in people with high trait impulsivity who are more sensitive to the sensitizing effects of substances (136) and potentially of stress. This hypothesis is consistent with elevated substance-cue-induced craving among people with a substance use disorder who have elevated trait impulsivity (256); elevated cue-induced craving, rather than high trait impulsivity, subsequently predisposed to relapse (257).

In addition, the transition from regular use to dependence and relapse could be marked by adaptations in stress and reinforcement mechanisms (45, 72, 73, 258). Koob presents a model in which stress and substances activate the HPA axis and induce the release of extrahypothalamic CRF which can stimulate the amygdala in people who use substances recreationally. This may mark the stages of experimentation and non-dependent regular use, and partly underlie positive reinforcement between drug and drug intake. By contrast, with prolonged and extensive use of substances the HPA axis becomes less activated by substances, and instead becomes activated when people are in a state of acute or prolonged withdrawal. This is accompanied by activation of the amygdala by extrahypothalamic CRF, creating a state of craving and compulsive want of a substance to diminish the accompanied negative emotional state (negative reinforcement). Brain systems entering this latter state may represent the transition from non-dependent to dependent use, and may be potentiated by stress, in which acute stress could elevate CRF and activate the amygdala, thereby accelerating transition to compulsive want for a substance (45, 72, 73, 258).

Finally, noradrenergic systems could become more sensitized, increasing the negative impact of stress-induced norepinephrine on impulsive responding (59), emotion, motivation, and control functions (54, 59, 60), and goal-directed actions (61), and increase acute bias for motivational information (62–64), suggesting an interaction with sensitized motivational systems. These effects have been related to the disrupting effects of norepinephrine on prefrontal functioning by flooding α1-adrenergic receptors (54, 55). This increase in sensitivity of noradrenergic systems was revealed by administering yohimbine, which mimics the stress response by antagonizing α2-adrenergic receptors, to people with cocaine (259), opioid (260, 261), or alcohol use disorder (262, 263). Yohimbine increased MHPG, the main metabolite of norepinephrine, and induced panic attacks in people with cocaine use disorder with 1–2 days of abstinence, but not in the same people when they had 2 weeks of abstinence (259). This suggests increased sensitivity of noradrenergic systems could diminish in 2 weeks of abstinence. However, although this may diminish effects on emotional systems, norepinephrine seems to remain sensitized and affect other systems suggested by yohimbine-induced increased startle in people with opioid dependence on methadone maintenance compared to healthy controls (260, 261), and increased startle in people with alcohol use disorder who had 2–4 weeks of abstinence who had more quit attempts (263).

**Figure 4:**
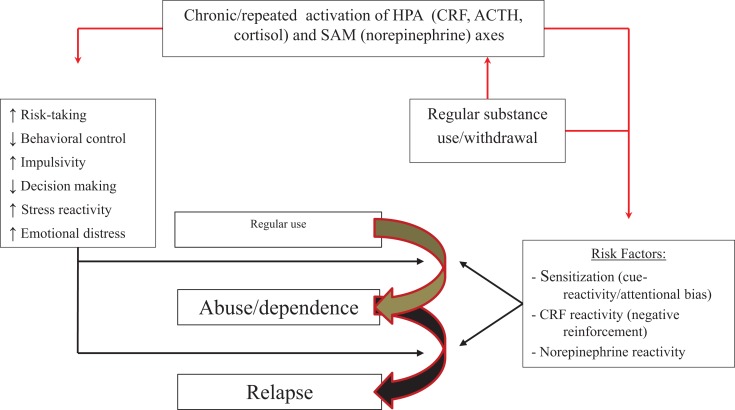
**Model of how stressors, in conjunction with chronic or repeated substance use, may affect progression from regular use to abuse or dependence**. Repeated activation of stress or motivational systems by stress and substances of abuse can (right side of figure) (1) intensify sensitization of motivational systems and enhance reactivity to and attentional bias for drug-related information; (2) increase HPA axis activation during acute withdrawal instead of substance intake and shift use from positively to negatively reinforcing; (3) increase sensitivity or reactivity of noradrenergic mechanisms and increase emotional distress to stress, impair control mechanisms, and enhance attentional bias or cue reactivity. These effects might operate in parallel with the effects of stress on trait-like factors (left side of figure). People with high trait impulsivity, who are more prone to sensitization, may be at particular risk.

Figure 4 presents the relationship between stress and chronic substance use, and development of dependence and risk of relapse. Three mechanisms may underlie stress- and substance-induced maintenance and relapse during dependence: intense sensitization of reward systems, which could remain sensitized for years after substance use (136), and which may occur primarily or more rapidly in people with high impulsivity combined with prior trauma or chronic or repeated stress; allostasis of stress mechanisms, driving use by negative rather than positive reinforcement; and elevated sensitivity of noradrenergic mechanisms, affecting control mechanisms. People more sensitive to sensitization or reactivity of stress systems may be at increased risk to transition to dependence and have more relapses under stress.

## Conclusion

### Stress and addiction illness-course

In this paper, we showed that childhood trauma and post-childhood chronic or repeated stressful events occur more frequently or are more chronic in people who use substances, and that trauma and prior or continued stressful events increase risk (1) to initiate use when provided with the opportunity to do so, and to initiate use at an earlier age; (2) to escalate use, and escalate use more rapidly; (3) to progress to compulsive use more rapidly; (4) to have lower motivation to quit; (5) to have faster or more frequent (re-)lapsed. These effects were generally found across classes of substances of abuse.

Table 1 summarizes relationships between a history of childhood trauma and stages related to addiction illness-course. Having experienced childhood sexual abuse or other serious trauma increases risk of (early) first use, facilitates transition to regular use, increases likelihood to develop dependence, and elevates risk to a lapse or relapse. Effects of childhood trauma are not limited to adolescence, but continue to exert their influence into adulthood. However, some outcomes are inconsistent, and data are missing on relationships between childhood trauma and severity of substance use disorders, motivation or likelihood to quit, and risk or severity of relapse. Although some studies looked at some of those variables, data for individual types of substances of abuse are limited.

**Table 1:** **Relationships between childhood trauma and stages of addictions divided by substance class**.

	Initiation		Regular use	Pathology		Quit	Relapse	
	Risk	Age	Risk	Risk	Severity	Odds	Risk	Severity
Nicotine	↑		↑	↑	↑*			
Alcohol	↑	↓/↔	↔	↑	↑*/↔		↔	
Marijuana	↑/↔	↔	↑	↑				
Cocaine	↑		↑	↔			↑**	↑**
Other stimulants	↑	↓	↑	↑				
Opiates	↑	↓	↑	↑				
Sedatives	↑	↓	↑	↑				
Hallucinogens								

↑, Increase; ↓, decrease; ↔, no effect; ↑/↔ or ↓/↔, inconsistent findings; blank cell, no data on individual types of substance. *Increased severity with a history of more trauma; **women only.

**Figure 5:**
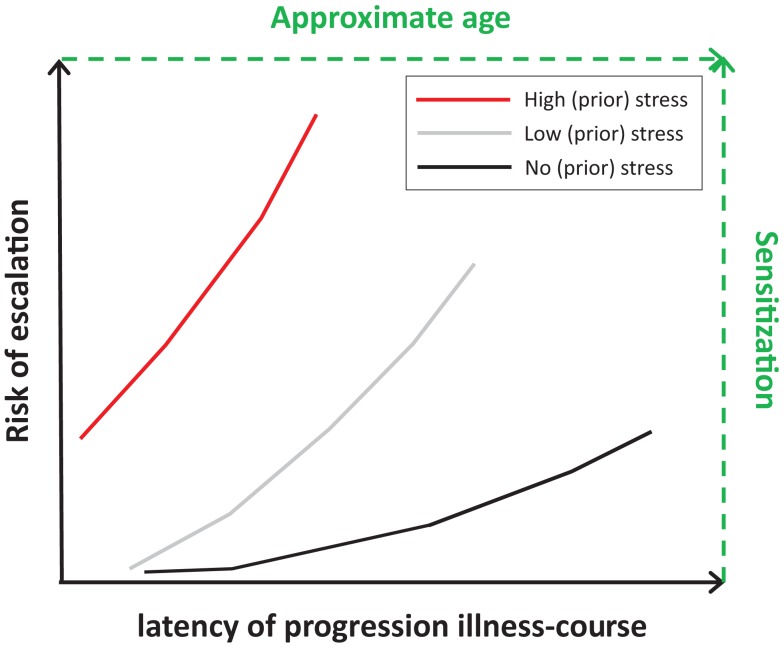
**Hypothesized relationships between no stress (black line), low stress (gray line), and high stress (red line) on risk and latency of escalation**. High stress relates to early onset and increased risk of (rapid) escalation to a substance use disorder (early onset and steeper risk-latency slope), whereas no stress relates to later onset and low risk to transition to problematic substance use or to a substance use disorder (later onset and shallow risk-latency slope). Effects of stress on risk and latency may follow those on level of sensitization of motivational systems and age of people (text in green).

Table 2 summarizes relationships between post-childhood chronic/repeated stressful events, including major stressful lifetime events or daily hassles, and stages related to addiction illness-course. Chronic or repeated stressful events have been related to increased risk to start use, to transition to regular use, to escalate to dependence, to lower chance to quit, and to lapse or relapse. Effects of stress on those stages might be mediated by negative affect or stress-induced increases in emotional distress or craving. However, information on transition to regular use and on relapse, in particular time to relapse or severity, is limited mostly to nicotine, alcohol, marijuana, and cocaine. Figure 5 presents the proposed relationship between risk of escalation of addiction illness-course and latency of escalation in people without and with childhood trauma or chronic/repeated post-childhood stressful events. People with repeated or severe trauma may be at the highest risk to start substance use early and escalate fast toward addiction, in particular with additional adulthood stress. Risk of escalation appears to follow sensitization of motivational systems, whereas latency to escalation may follow age.

**Table 2:** **Relationships between stressful events and stages of addictions divided by substance type**.

	Initiation		Regular use		Pathology		Quit	Relapse	
	Risk	Age	Risk	Severity	Risk	Severity	Odds	Risk	Severity
Nicotine	↑	↓*	↑/↔	↑	↑	↑/↔	↓	↑	
Alcohol	↑	↓/↔	↑	↑/↔	↑	↑/↔		↑/↔	
Marijuana	↑	↓	↓		↑	↑	↓	↑	
Cocaine	↑	↓			↑	↑		↑
Other stimulants	↑	↓							
Opiates	↑	↓						↔	
Sedatives	↑	↓							
Hallucinogens	↑	↓							

↑, Increase; ↓, decrease; ↔, no effect; ↑/↔ or ↓/↔, inconsistent findings; blank cell, no data on individual types of substance; *girls only.

### Potential underlying mechanisms

Stress and substance of abuse share common mechanisms, activating stress and reward systems. Stress puts people at risk to use substances and develop a substance use disorder, and substance use put people at risk for increased stress reactivity and more stressful events.

**Figure 6:**
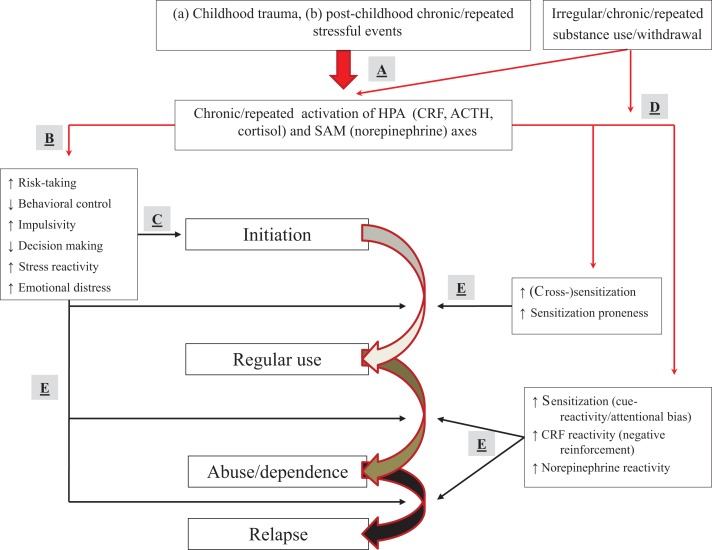
**Full model of how stressors and substance use may affect progression from experimentation to dependence and relapse**. **(A)** Childhood trauma or chronic/repeated post-childhood stressful events (as well as substances) activate, and change activation patterns of the HPA and SAM axis. **(B)** Stress affects trait-like factors (elevating risk-taking, impaired decision making, and behavioral control) or sensitization mechanisms (dopaminergic motivation, amygdalar CRF, and norepinephrine), predisposing people to use substances. **(C)** Stress effects on trait-like factors can result in first-time use of a substance. **(D)** Stress and irregular, repeated, or chronic substance use can affect sensitization mechanisms. **(E)** Sensitization (in conjunction with stress effects on trait-like factors) can result in escalation to regular use, abuse/dependence, and lapse/relapse. People with trait-like high risk-taking, impaired decision making, poor behavioral control or high impulsivity, increased stress reactivity, or elevated emotional distress with a belief that substances diminish negative affect, are at particular risk.

We presented potential mechanisms related to the effect of stress on vulnerability to use substances and progress to the next addiction illness-course stage. These mechanisms might cut across childhood trauma and post-childhood stressful events, as well as across classes of substances of abuse. The full model is presented in Figure 6.

The effect of stress on initiating substance use might relate to stress-induced enhanced risk-taking, impaired decision making, and poor behavioral control. At particular risk may be individuals with trait-like increased stress reactivity, increased risk-taking, poor decision making, impaired behavioral control or higher impulsivity, and enhanced emotional distress with a belief system that substances can diminish negative affect.

Progression to regular use may be related to stress-induced cross-sensitization of reward systems to motivational effects of substances of abuse, increasing the motivational value of lower doses of substances, inducing an earlier shift to regular use, and escalating the dose and speed of intake. This mechanism may operate in parallel with the trait-like factors described above. Sensitization could be modulated further by irregular substance use. At particular risk may be individuals with high trait impulsivity who are more prone to sensitization.

Finally, the transition to heavy use, dependence, and risk for relapse may relate to stress- and substance-induced intensified sensitization of reward mechanisms, allostatic changes in stress systems and their relationship with amygdala-mediated negative reinforcement, and increased sensitivity or reactivity of noradrenergic systems. These mechanisms may operate in parallel with trait-like factors described above. Also here, at particular risk may be individuals with high trait impulsivity who are more prone to sensitization.

### Limitations

Outcomes across studies are inconsistent regarding which stressors might pose the most severe risk to escalate illness-course, although more chronic emotional stressors such as financial problems seem to have more pronounced effects. Second, the literature is mostly aimed at alcohol, smoking, marijuana, and cocaine, with more restricted information for other types of substances. Third, information is more restricted for some than for other stages of the addiction cycle, in particular the phase associated with deciding or trying to quit. Finally, it is sometimes difficult to decide what stages studies focused on due to limited information on symptom severity among people using substances on a recreational basis.

## Author Contributions

Marijn Lijffijt developed the concept of this paper, did literary searches, and wrote the first draft. Kesong Hu and Alan C. Swann have contributed significantly to interpreting the literature and critically read and revised earlier drafts.

## Conflict of Interest Statement

The authors declare that the research was conducted in the absence of any commercial or financial relationships that could be construed as a potential conflict of interest.
